# Evolutionary transgenomics: prospects and challenges

**DOI:** 10.3389/fpls.2015.00858

**Published:** 2015-10-20

**Authors:** Raul Correa, David A. Baum

**Affiliations:** ^1^Department of Molecular and Human Genetics, Baylor College of MedicineHouston, TX, USA; ^2^Department of Botany, University of Wisconsin-MadisonMadison, WI, USA

**Keywords:** developmental system drift, evolution, evo-devo, genetic screens, speciation genes, transgenomics, transformation

## Abstract

Many advances in our understanding of the genetic basis of species differences have arisen from transformation experiments, which allow us to study the effect of genes from one species (the donor) when placed in the genetic background of another species (the recipient). Such interspecies transformation experiments are usually focused on candidate genes – genes that, based on work in model systems, are suspected to be responsible for certain phenotypic differences between the donor and recipient species. We suggest that the high efficiency of transformation in a few plant species, most notably *Arabidopsis thaliana*, combined with the small size of typical plant genes and their *cis*-regulatory regions allow implementation of a screening strategy that does not depend upon *a priori* candidate gene identification. This approach, transgenomics, entails moving many large genomic inserts of a donor species into the wild type background of a recipient species and then screening for dominant phenotypic effects. As a proof of concept, we recently conducted a transgenomic screen that analyzed more than 1100 random, large genomic inserts of the Alabama gladecress *Leavenworthia alabamica* for dominant phenotypic effects in the *A. thaliana* background. This screen identified one insert that shortens fruit and decreases *A. thaliana* fertility. In this paper we discuss the principles of transgenomic screens and suggest methods to help minimize the frequencies of false positive and false negative results. We argue that, because transgenomics avoids committing in advance to candidate genes it has the potential to help us identify truly novel genes or cryptic functions of known genes. Given the valuable knowledge that is likely to be gained, we believe the time is ripe for the plant evolutionary community to invest in transgenomic screens, at least in the mustard family Brassicaceae where many species are amenable to efficient transformation.

## Introduction

At its most general, evolutionary developmental biology, “evo-devo,” seeks to understand how development, the translation of a genotype to a phenotype in a given environment, constrains, or enables phenotypic evolution (Stern, 2000; Wagner et al., 2000; Arthur, 2002; Carroll et al., 2004; Lee et al., 2014). The core data that are needed to achieve such understanding are genetic and developmental changes that have been shown experimentally to cause particular evolutionary transitions from ancestral to derived phenotypes. While much evo-devo research can focus on phenotypic variation within living populations, there has long been an interest in also studying characters that differ between living species, where one species manifests the ancestral character state and the other manifests the derived. How, then, can we experimentally determine the genetic and developmental basis of traits that differ between species?

Until now, the search for genes responsible for species differences has mainly exploited either candidate gene or quantitative trait locus (QTL) approaches. Candidate gene methods use information from genetic model systems to hypothesize that a certain genetic change caused the transition from the ancestral to the derived character state and then set about to test that hypothesis using comparative studies of two (or more) species that differ for the trait. The test usually involves comparative expression studies combined with various functional studies, which might include knocking down gene expression and/or moving the candidate gene among species either by crossing or transgenic methods. However, candidate gene approaches are limited to phenotypes whose developmental basis is well understood in model genetic systems. While much of what we know about evo-devo comes from candidate gene studies, they have limitations. In particular, when a phenotype is caused by unpredictable genes, as can happen due to neofunctionalization [e.g., (Vlad et al., 2014)], candidate gene approaches will come-up empty. Indeed, using only a candidate gene approach would make it very hard to answer one of the key questions in evo-devo: how many different genetic pathways are available for the evolution of a new trait?

Currently, the main alternative to candidate gene approaches is QTL analysis. This involves crossing two species with contrasting traits and then looking for cosegregation of the trait with genetic markers in the F2 or later generations. The goal is to positionally clone genes causing phenotypic differences and, eventually, home in on the sequence difference causing the trait difference. However, QTL analysis is limited to cases where species are capable of being crossed. Further, it is notoriously difficult to clone the gene underlying a QTL in non-model species.

In this paper we will argue that evolutionary transgenomics (Baum, 2002) represents a third method, complementary to the other two, that could be used to identify the genes responsible for species’ differences. An evolutionary transgenomic screen involves introducing genomic fragments of a donor species into the genome of a recipient species (or perhaps a divergent ecotype) and screening the resulting transgenic lines for phenotypic effects. Such screens have the advantage of not being limited to crossable species and yet being able to find genes that would not have been predicted *a priori*. We will suggest that while transgenomics poses practical challenges, it has great potential value for plant evo-devo research in those taxa that are readily transformed and may help us identify many genes of evolutionary and developmental interest that would otherwise be difficult to discover.

Systematic screens in yeast, including screens of plant cDNA libraries, have successfully been used to identify genes controlling cell-level phenotypes [e.g., (Papoyan and Kochian, 2004; Liu et al., 2007)]. However, there have been few attempts to screen the genome of one multicellular eukaryote in that of another. Because there are many plants that are closely related, show many phenotypic differences, have compact genes, and are readily transformed, plants are better suited to transgenomics than are most animals. But are the benefits to be gained likely to outweigh the work entailed in conducting evolutionary transgenomic experiments? In this paper we use evolutionary theory and data from prior interspecies transformation experiments and our published, pilot transgenomic screen (Correa et al., 2012) to assess the approach and how it could best be implemented. We conclude that the time is ripe to develop transgenomics resources at least in Brassicaceae Burnett, a clade of flowering plants, many of whose species can now be transformed with high efficiency.

## Principles Of Transgenomics

In an evolutionary transgenomic screen, fragments of genomic DNA from a donor species are added to the wildtype genome of a recipient species. We then screen primary transformants (T1s) to look for phenotypic effects that might be due to the inserted DNA. Since plant transformation usually entails inserting an extra piece of DNA rather than homologous replacement of endogenous sequences, inserts will only cause phenotypes in T1s if they act in a *transdominant* manner. That is to say, one copy of the foreign gene must manifest a phenotypic effect even in the presence of two functional copies of that gene (if any exist in the recipient genome). Genetic theory suggests two primary causes of a *transdominant* phenotype.

The first potential cause of a *transdominant* phenotypic effect is the addition of a supernumerary gene copy to the genome, a gene dosage effect. It is well documented that changes in gene dosage can have phenotypic effects [reviewed by (Birchler and Veitia, 2007)]. This is most obvious when aneuploids (e.g., trisomics) yield distinct phenotypes, including lethality or sterility. Dosage effects presumably result from additional copies altering the balance of expression of genes in regulatory pathways. Addition of a single additional gene (as would occur in a hemizygous T1 transgenic plant) might be expected to increase expression level by approximately 50%, or higher if multiple insertions of the transgene occur. However, the actual effect on expression will vary subject to position effects and whether or not transgene silencing is triggered.

It is not known how often dosage alone will yield a visible phenotype in a transgenic line, and this might vary depending on the phylogenetic distance between donor and recipient species. However, based on prior transgenic data it seems likely that many dosage effects will primarily be quantitative. For example, it has been found that adding extra *Arabidopsis thaliana* (L.) Heynh. *LFY* transgenes to a wildtype *A. thaliana* background has a dosage dependent effect on flowering time (Blazquez et al., 1997).

Dosage effects do not depend on sequence divergence between donor and recipient species. Quite the contrary – a dosage effect depends upon conservation of molecular function between the endogenous and exogenous gene copies. This leads to a powerful test for discriminating dosage effects from other mechanisms: if the phenotypic effect can be replicated by introducing the homologous fragment of the recipient species back into the recipient species, then dosage is likely to be the cause of the observed phenotype. If, there is no homologous region, or if the homologous region fails to cause the phenotype, then dosage is unlikely to be responsible.

**FIGURE 1:**
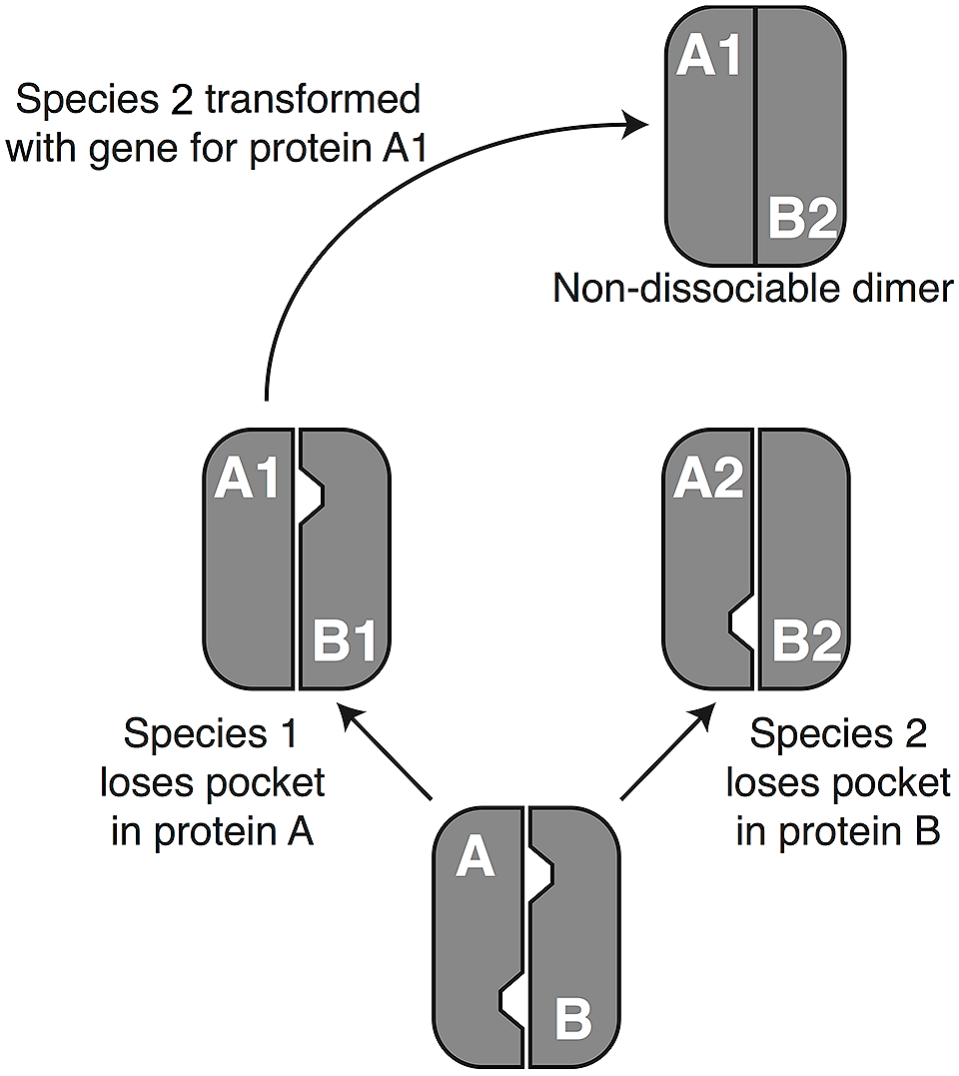
**A possible mechanism of development system drift (DSD).** DSD occurs when two lineages remain phenotypically unchanged but undergo genetic divergence (True and Haag, 2001). The phenomenon depends primarily on evolutionary changes that influence the way that proteins interact with each other and with DNA sequences (Haag, 2007; Landry et al., 2007), although changes in miRNA’s and their targets (Mallory et al., 2004) have a potential role as well. To understand how such phenomena can lead to a dominant phenotype in a transgenomic screen, consider a hypothetical example in which two proteins, A and B, dimerize but need to dissociate during normal development. In a hypothetical ancestor, pockets in both proteins destabilize the dimer enough to permit dissociation. In the lineage leading to species 1, protein A (A1) loses its pocket, but dissociation is still achieved thanks to the pocket in protein B1. Conversely, the pocket in protein B2 has been lost on the lineage leading to species 2. Moving protein A1 in species 2 (or B2 into species 1) will cause formation of a non-dissociable dimer, resulting in a dominant disruption of normal development. When development is disrupted sufficiently to cause inviability or sterility, DSD can enforce reproductive isolation between lineages, because hybrids do not survive to reproduce. In that case the pattern conforms to the Dobzhansky–Muller model of speciation [e.g., (Bomblies et al., 2007; Landry et al., 2007)], showing that transgenomics offers a novel way to identify genetic interactions that could contribute to speciation.

The second potential cause of a *transdominant* phenotype is evolutionary divergence between the donor and recipient genes. This could arise through one of two mechanisms, developmental system drift (DSD) or phenotypic divergence. DSD occurs when proteins and/or regulatory DNA/RNA sequences coevolve without altering visible phenotypes (True and Haag, 2001). The “drift” in underlying molecular mechanisms can cause a gene from the donor species to malfunction in the recipient genome in a such a way that a phenotype is seen, analogous to transgressive segregation, which is often seen in QTL studies (Rieseberg et al., 1999). To make the concept more concrete, **Figure 1** shows a hypothetical example involving a protein with two subunits that must be disassembled for proper development, with disassembly requiring at least one “pocket” of low attraction between the two subunits. Reciprocal loss of the pocket in the two subunits could result in a case in which subunit A from species 1 yields a dominant-negative phenotypic effect when placed in the genome of species 2. It is worth noting the similarities between this DSD model and Dobzhansky–Muller interactions (Kondrashov et al., 2002; Bomblies et al., 2007). Indeed, one exciting aspect of transgenomics is its potential to identify potential hybrid inviability genes.

**FIGURE 2:**
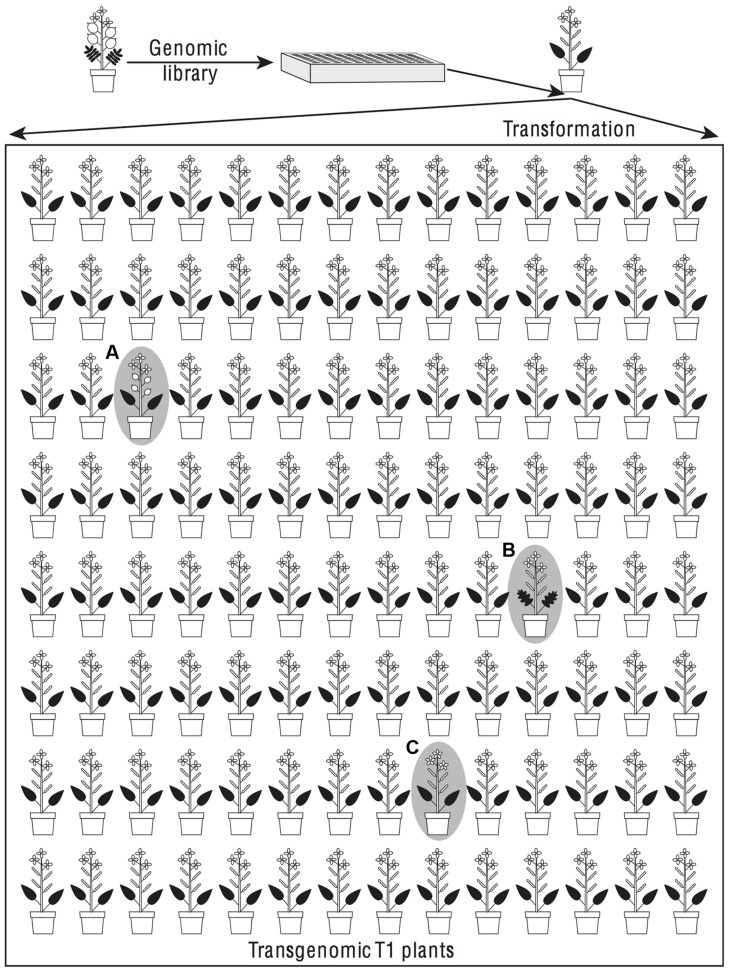
**Dominant phenotypic effects found in a transgenomic screen could reflect phenotypic divergence or developmental system drift between donor and recipient species.** In a transgenomic screen random genomic fragments from a donor species are introduced by transformation into a recipient species. The transformants are screened for phenotypes that differ from the recipient species. When an insert causes a phenotype of the donor species to be found, for example round, flattened fruits **(plant A)**, it is likely that the transgene contains a major gene contributing to the evolution of that phenotype. If the phenotype is intermediate between the donor and recipient species, for example pinnately lobed **(plant B)** rather than either entire or pinnately compound leaves, then the transgene is a candidate for being one of several genes that changed during phenotypic divergence. If the phenotype resembles neither the donor nor the recipient species, as for example in having five rather than four petals **(plant C)**, then developmental system drift is suggested.

**FIGURE 3:**
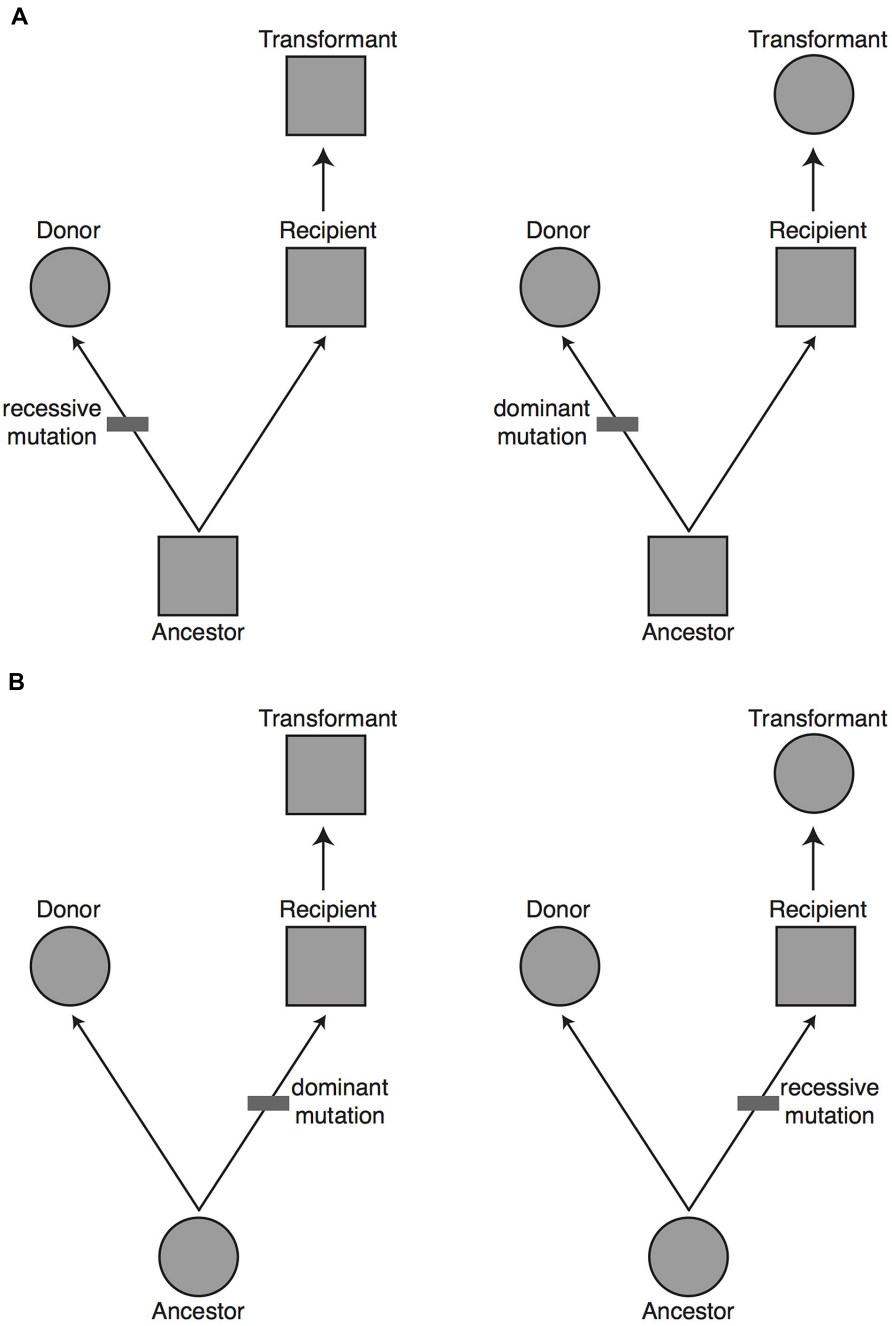
**The proportion of major genes expected to be uncovered in a transgenomic screen.** Based on evolutionary principles we would predict that 50% of the major genes responsible for a phenotypic difference between the donor and recipient species will be found in a unidirectional transgenomic screen. To see why this is so, imagine a potential donor and recipient species for a transgenomic screen that differ in a phenotype (circle vs. square, respectively). Without further information it is equally likely that: **(A)** the donor species has the derived phenotype, with a change having occurred on the lineage from the common ancestor to the donor species (upper panels), or **(B)** the donor has the ancestral phenotype, with a change having occurred on the lineage from the common ancestor to the recipient species (lower panels). The mutation that gave rise to the derived phenotype could have been fully recessive or at least partially dominant. If the donor has a derived phenotype that is dominant (top right), or it has an ancestral phenotype that is dominant (bottom right), then moving the causal gene into the recipient will yield a dominant phenotype. In approximately 50% of cases (left panels) the causal gene will not yield a dominant phenotype when moved from the donor to the recipient. The major genes missed in a unidirectional transgenomic screen could theoretically be found with a reciprocal screen in which the genome of the former recipient species is screened in the background of the former donor species.

The alternative explanation of a *transdominant* phenotype is that phenotypic evolution has been driven by sequence evolution (whether in coding or regulatory regions) at genes of large effect. In this case, a gene can carry the donor species’ phenotype into the recipient species’ genome (**Figure 2**). This is expected to happen when a difference in phenotype is due to a fully or partially dominant mutation on the lineage leading to the donor species or to a recessive mutation on the lineage leading to the recipient species (**Figure 3**). Assuming that the relative frequency of dominant and recessive mutations is about equal on the two evolutionary lineages, first principles would suggest that a complete transgenomic screen would be able to detect about 50% of the major genes explaining phenotypic differences between the donor and recipient species.

If we knew how many of the phenotypic differences between species were due to genes of large effect we could predict the frequency with which transgenomic lines will manifest a phenotype that resembles the donor species. Unfortunately, we are not aware of any relevant quantitative data. Indeed, one of the most compelling reasons to conduct transgenomic screens is because they will help quantify the frequency of evolution via genes of large effect, something that has long been a source of controversy (Gottlieb, 1984; Doebley and Lukens, 1998; Hoekstra and Coyne, 2007).

## Alternative Transgenomic Strategies

**FIGURE 4:**
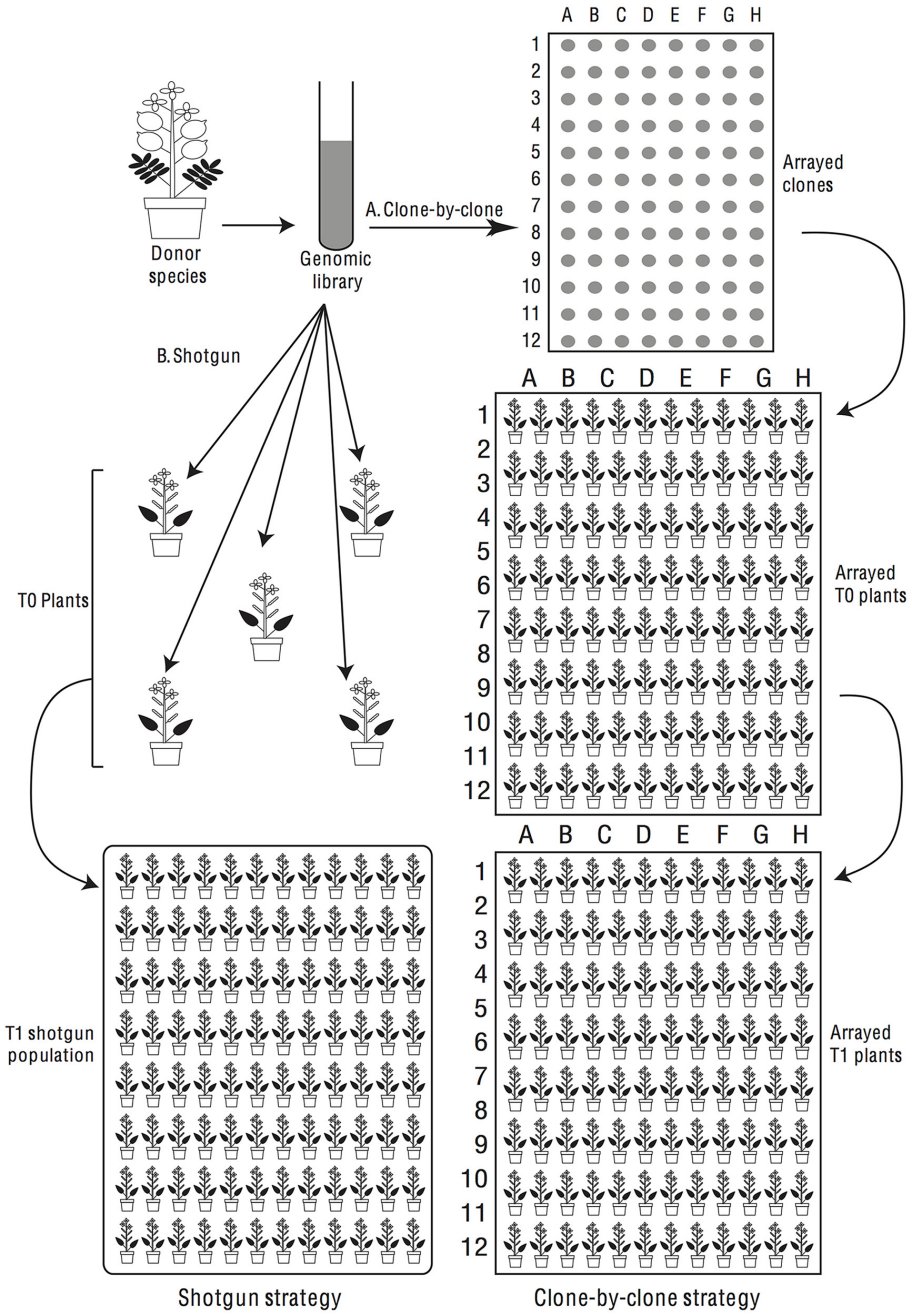
**Donor species genes can be screened for phenotypic effects in the recipient species by shotgun or clone-by-clone strategies.** In a shotgun screen (left half of flow-chart) the library is transferred *en masse* into recipient plants, which are screened phenotypically. In a clone-by-clone screen (right half of flow-chart), clones are arranged in microtiter plates and transformants are generated for each isolated clone.

In considering transgenomics two main experimental approaches suggest themselves. One approach is a shotgun strategy, where we generate a genomic library of a donor species in a suitable bacterium, e.g., *Agrobacterium tumefaciens* (Smith et Town.) Conn, and then introduce this *en masse* (or perhaps in pools) into a population of recipient plants (**Figure 4**). T1s would be screened for phenotypes of interest and, when a phenotype is observed, we would determine *post hoc* what insert had been introduced into the recipient’s genome. Alternatively, a clone-by-clone strategy can be followed in which we isolate individual clones from a genomic library and use each clone for multiple transformations of the same recipient species to identify repeatable phenotypic effects (**Figure 4**).

A shotgun strategy has the advantage of quickly generating large populations of transformant plants to screen for phenotypes of interest. Nonetheless, it does have some serious drawbacks. (1) When interesting phenotypes are seen it might not be trivial to isolate the responsible genomic fragment, especially for large inserts or for inserts that cause sterility meaning that one could not obtain much transgenic plant tissue. (2) Because each clone will be introduced into only one recipient, a phenotypic effect could be a false positive due to genetic or microenvironmental differences among transformed plants. (3) Clones that cause dominant early lethality will not be identified so their frequency in the genome could not be assessed. (4) A shotgun-transformed pool could not readily interface with existing genomic information to yield a durable resource for other researchers to utilize.

A clone-by-clone strategy has the virtue that the identity of inserts is easily determined (by sequencing the clone) and one can obtain multiple T1 plants per clone, reducing the false positive problem. Furthermore, inserts causing early lethality can, at least theoretically, be identified by their inability to generate mature transgenic plants and, once a population of transgenomic lines has been made, it represents a durable resource that could be screened repeatedly for different phenotypes in a diversity of growth conditions. On the other hand, a clone-by-clone strategy requires more work to separate and bulk-up individual clones and requires one to do many more individual plant transformations.

Whether one uses a shotgun or clone-by-clone approach, once transgenomics lines are found to have phenotypic effects a number of different downstream experiments can be undertaken. Before, or in parallel with, standard methods for studying gene-function (e.g., isolation of T-DNA insert lines, double mutant assays, expression studies) experiments should be conducted to assess the role of sequence divergence between donor and recipient species in explaining the phenotype. Some critical experiments will include: (1) repeating transformation to confirm that the phenotype is caused by the insert; (2) subcloning the insert to identify the causal gene region; (3) introducing the homologous gene from the recipient species as an extra copy to assess if the result is due to gene dosage; (4) generating chimeric constructs between the donor and recipient genes to locate the causal differences, and; (5) isolating the homologous gene from additional species to assess the correlation between the phenotype of the donor species and the ability of the gene to confer an effect in transgenic lines of the recipient species. Through such experiments, there is every reason to hope that we could eventually arrive at a clear understanding of the molecular and developmental basis of the transgene’s effect on phenotype and, in some cases, shed light on the evolution of phenotypic differences between species.

## A Pilot Clone-By-Clone Screen

Correa et al. (2012) piloted a clone-by-clone transgenomic screen using genomic clones with ∼20 kb inserts from the gladecress *Leavenworthia alabamica* Rollins introduced into *A. thaliana*. *Leavenworthia alabamica*, and *A. thaliana* differ in almost all visible morphologies and yet they are both members of Brassicaceae Lineage I (Beilstein et al., 2008, 2010; Franzke et al., 2011). While this was a large experiment, high-throughput “drip” transformation allowed one graduate student and 2–3 undergraduate assistants to screen T1s for as many as 750 clones per month.

Of the 1134 *L. alabamica* clones screened, 84 produced an initial T1 that deviated from “normal”. However, in only eight cases was the initial “abnormal” phenotype repeated in additional independent T1s. Correa et al. (2012) focused on one clone that was shown to cause stunted fruit and increased seed abortion in a *transdominant* manner. Sequencing of the clone insert suggests that this effect is most likely explained by a gene region that shows homology to the *A. thaliana SLOW-WALKER2* (*SWA2*) gene. *SWA2* encodes a protein that is important for ribosome biogenesis, and *A. thaliana swa2* mutants manifest shortened fruit and seed abortion (Li et al., 2009). Follow-on experiments have confirmed that a subclone containing the region of *SWA2* homology is sufficient to cause these phenotypes and have detected *L. alabamica SWA2-like* mRNA in developing fruit (Wu and Baum, unpublished data).

What lessons were learned thanks to this pilot transgenomic screen? On the plus side, we established that a clone-by-clone strategy is feasible, at least when *A. thaliana* is the recipient, and showed that it is possible to use this strategy to identify a gene that can alter the phenotype of a recipient in a *transdominant* manner. Back-of-the-envelope calculation would suggest that if 750 clones can be screened per month by a small team piloting the approach for the first time, a larger and more experienced team could realistically screen more than 10,000 clones per year making it plausible that one could screen a moderate size genome to saturation.

## Could Transgenomics Identify Major Genes Explaining Species Differences?

The single gene identified by Correa et al. (2012) is likely to reflect DSD rather than phenotypic divergence. The question this raises is whether this result indicates that the method has limited utility for scientists whose goal is to identify genes that explain species differences.

The first fact to emphasize is that the screen conducted by Correa et al. (2012) covered less than 5% of the donor species’ genome. Furthermore, considering that with a false negative rate of at least 50%, only 2.5% of the donor genome was effectively screened. This means that it would be grossly premature to take the results of Correa et al. (2012) as indicating that transgenomics cannot find species-differentiating genes. Thus, the best we can do is to look at prior experiments in which single genes have been moved between closely species to evaluate how often we might expect to find a dominant phenotypic effect in a transgenomic screen that is indicative of some functional role in explaining species differences (Correa et al., 2012).

A majority of experiments in which a gene (coding region or cDNA) from one species is moved into a different species use either a broadly active promoter such as 35S or the homologous promoter from the recipient species. The most common result from such studies is that the exogenous gene functions equivalently to the endogenous gene [e.g., (Whipple et al., 2004; Maizel et al., 2005; Dornelas and Rodriguez, 2006; Busch and Zachgo, 2007)]. Sometimes, especially with distantly related donors, the exogenous gene shows reduced functionality resembling a partial loss-of-function allele [e.g., (Tzeng and Yang, 2001; Maizel et al., 2005)]. Some studies using 35S have combined transformation data with evidence on comparative gene expression to show that altered expression of a single functionally conserved gene likely contributed to the evolution of plant phenotypes (Doczi et al., 2005; He and Saedler, 2005; Lee et al., 2005; Maizel et al., 2005; Zahn et al., 2005; Hay and Tsiantis, 2006; Busch and Zachgo, 2007; Hovav et al., 2007). In one case, the dominant effect of a full-length transgene was demonstrated using introgression, rather than transformation (Hovav et al., 2007). Likewise, some experiments using 35S have yielded novel phenotypes, with examples including pathogen resistance (Lee and Lee, 2005; Lee et al., 2005; Zahn et al., 2005; Carlson et al., 2006), stress-tolerance (Hsieh et al., 2002; Liu et al., 2007; Wang et al., 2007), the production of novel secondary metabolites (de Majnik et al., 2000; Mietkiewska et al., 2004; Mori et al., 2004), and even donor species-like morphology (Fourquin et al., 2013) that hint at evolutionary divergence in protein function sufficient to cause a dominant phenotype in a transgenomics screen.

Another class of interspecies transformation experiments move homologous *cis*-regulatory (“promoter”) regions with reporters from one species to another to see if expression patterns are conserved [e.g., (Lin et al., 1993; Burger et al., 2006)] or divergent [e.g., (Doczi et al., 2005; Hay and Tsiantis, 2006)]. When a donor species’ promoter drives expression in developmental stages or tissue types where the recipient species’ promoter is inactive [e.g., (Zahn et al., 2005; de Martino et al., 2006)] there is a potential for a dominant phenotypic effect to be found in a transgenomics screen.

More direct evidence comes from those few studies that have moved genomic fragments with both *cis*-regulatory and coding regions between species. One example comes from work on the evolution of self-compatibility in *A. thaliana*. Genomic fragments from the S-locus of the self-incompatible *A. lyrata* (L.) O’Kane et Al-Shehbaz, when used to transform wildtype plants of the self-compatible *A. thaliana*, converted the latter to self-incompatibility – the effect varying with ecotype of the recipient plants (Nasrallah et al., 2002, 2004). This effect appears to be due to divergence in the coding region (loss of gene function in *A. thaliana*) rather than the evolution of regulatory regions. Similarly, Vlad et al. (2014) used transformation to show that the presence of the gene *REDUCED COMPLEXITY* (*RCO*) in the genomes of *A. lyrata* and *Cardamine hirsuta* L., but its loss in *A. thaliana*, largely explains the dissected leaf shape of the former species and the simple leaves of the latter. Another example involves the introduction of *LFY* and *TFL1* genes from different Brassicaceae species into *A. thaliana*. In all three cases involving *LFY* (Yoon and Baum, 2004; Sliwinski et al., 2007) and the one case involving *TFL1* (Liu et al., 2011), the transgene resulted in a novel phenotype and these were shown to also occur in a wildtype background showing transdominance (Sliwinski et al., 2006, 2007; Liu et al., 2011). Similarly, studies of *REPLUMLESS* (*RPL*), a gene that promotes fruit dehiscence, showed that introducing the *Arabidopsis RPL* gene into *Brassica* is sufficient to induce *Arabidopsis*-like fruit dehiscence in the recipient (Arnaud et al., 2011). As was also found in the *LFY* and *TFL1* experiments, the transgene effect is not replicated when using the endogenous gene copy, showing that the effect is due to sequence divergence (specifically in *cis*-regulatory regions) rather than to a gene dosage effect.

Taken together, evolutionary theory and prior candidate gene interspecies transformation experiments suggest that a transgenomics screen has a high potential to uncover *transdominant* phenotypic effects. Given this we should assess how best to design future transgenomic screens to maximize their efficiency.

## Practicalities Of Screening

One of the striking findings of Correa et al. (2012) was the high false positive rate. Specifically, out of 84 cases where an abnormal phenotype was seen in an initial T1, only eight recurred in further T1s from the same clone, and only one was definitively shown to be due to a *L. alabamica* insert. It is not surprising that false positives arise since there is likely to be genetic variation among the plants used for transformation and even wildtype plants occasionally manifest phenotypic abnormalities (Hempel and Feldman, 1995). Furthermore, transformation and *Agrobacterium* infection are both potentially mutagenic, so some abnormalities likely reflect *de novo* mutation. However, although some false positives are inevitable, it is worth considering strategies for reducing their frequency so as to avoid wasting time and effort following up phenotypes that are not caused by the transgene.

One strategy that we have explored is to immediately grow-up and screen ∼5 T1s per clone instead of just one. Once one is screening seedlings on plates for a selective resistance trait, it is not much more difficult to transplant and retain five rather than one T1. Requiring that phenotypes recur in at least 2–3 of the independent T1s from the same clone would go a long way toward excluding phenotypic effects that are due to a position effect or an insertional mutation in an endogenous gene, since independent T1s are not expected to have their inserts integrated at the same *A. thaliana* locus. Furthermore, if the locations of plants used to test a particular clone are randomized within growth rooms, there is little chance that multiple T1s would have the same phenotype because of a shared microenvironment.

An additional benefit of screening several T1s per clone is that it should also reduce the number of false negatives: overlooking a causally important clone due to a lack of a visible phenotype in the initial T1 screened. Despite clear evidence that the clone containing the *SWA2*-like region causes increased seed abortion and reduced fruit size, Correa et al. (2012) noted that about one half of the T1s containing the clone showed a wild type morphology. This matches other experiments that have reported that many clones fail to manifest a phenotype in *A. thaliana* due to transgene silencing (Morel et al., 2000; Schubert et al., 2004). However, while there are benefits to screening several rather than just one T1, such an approach significantly increases the space needed to conduct the initial screen. So, if space limitations rather than labor limitations are paramount, this strategy might not be worth deploying.

We have also tried a further embellishment to reduce the impact of genetic differences among the transformed (T0) plants. This involved growing ∼5 independent T1 plants germinated as usual on selective agar plates and in parallel growing the same number of plants from the same seed stock but isolated from non-selective plates. Based on *Arabidopsis* transformation efficiency, non-selectively grown plants are much (ca. two orders of magnitude) more likely to lack donor species DNA than to contain it. This means that phenotypic effects that recur in the T1 plants but are absent in non-selectively grown siblings are much more likely to be due to a *transdominant* donor species gene. However, this represents a further doubling of the space required and additional work for making plates, transplanting seedlings, and scoring plants for abnormalities. Based on our informal experimentation, we are doubtful that this additional work would yield sufficient benefits to make it worthwhile.

## Choice Of Donor And Recipient Species

Whichever transgenomic strategy is used, the choice of donor and recipient species is important. As the preeminent plant genetic model system, blessed with efficient transformation methods, *A. thaliana* is the ideal recipient species with which to initially implement and assess a transgenomics approach. While other readily transformed plant species such as rice, tobacco, and petunia are worth considering, we believe that the efficiency of dip-transformation makes Brassicaceae the clade of choice in which to first try transgenomic research.

The strengths of transgenomics would be greatest when working with a donor species that is closely related to the recipient so that many of the core developmental process and genes are shared, but distant enough that many visible phenotypes differ. Alternatively, if one is committed to a particular trait (e.g., salt tolerance, metal hyperaccumulation, leaf shape, etc.), one can pick a donor species that differs from *A. thaliana* in at least that phenotype. What we do not know is how far from the phylogenetic neighborhood of *A. thaliana* you can go before most phenotypic effects are hard to make sense of: *Brassica* L., *Cleome* L., papaya, cotton, tobacco, rice, moss?

An additional consideration is the availability of genetic tools in the donor species. If the donor genome has been sequenced it will be that much easier to home in on causal regions once an interesting phenotype has been found. Also, if the donor species is one that can be transformed then it will be possible to introduce *A. thaliana* genes, which may be useful for exploring gene function. Furthermore, if both species can be transformed with high-throughput methods, it becomes possible to undertake a full, bidirectional transgenomics screen, which should allow one to identify almost all genes of large effect that have contributed to the phenotypic divergence of the two species (**Figure 3**).

## Cloning Strategy

Once the donor and recipient species have been identified, a number of detailed practical issues will need to be addressed, many of which could significantly influence the efficacy of the screen. Among these is the strategy used to assemble a genomic library, most notably insert size, choice of vector, selectable marker, and possible target-enrichment strategy.

Large inserts will presumably allow for more rapid screening of a genome to completion. A further advantage of long inserts is they are less likely to include truncated genes, which can yield false positive results if, for example, a truncated donor species protein binds to and prevents a partner protein from interacting normally with the homologous recipient species protein (Villagarcia et al., 2012) or if the transgene region is missing a negative regulatory protein domain or a *cis*-repressor element. On the other hand, longer clone inserts are more difficult to work with in the lab and typically show lower transformation efficiencies. Furthermore, inserts that are too large to allow amplification from T-DNA primers will make it markedly more difficult to isolate the insert sequence from transformant plants, as might be necessary for a shotgun transgenomic screen.

Vectors should be ones that can achieve sufficient transformation efficiency for inserts of the target size, yet should tend to yield only one insert per transgenic line so as to minimize confounding dosage effects. Additionally, all things being equal, it might be helpful to engineer a vector that results in inserts being flanked by insulator sequences (She et al., 2010; Singer and Cox, 2013). By reducing the extent to which gene expression is affected by where in the genome an insert lands, insulator sequences might reduce line-to-line variability and, thus, lower the false negative rate.

The choice of selectable marker will be guided by speed of screening as well as the desirability of being able to identify transgenic lines at a very early developmental stage so that genes causing early lethality can be found. For example, it might be possible to use a selectable marker that causes embryos to fluoresce (Ali et al., 2012), allowing transformants to be visually identified as seeds and then grown up, non-selectively, on soil.

The final factor to consider in designing a transgenomic screen is whether it might be possible to manipulate the genomic library to increase the proportion of clones that includes potentially causal genes. While transgenomics is premised on the idea that we want to focus on genes with their native *cis*-regulatory machinery, meaning that we must include abundant non-coding content in our inserts, there is certainly much of the genome that is, *a priori*, less likely to cause informative *transdominant* phenotypes in a foreign genome. Our initial focus might be to enrich for the gene-space or, if we are focusing on a particular phenotype, we might be specifically interested in enriching for genes that are expressed in a particular organ. There is a diversity of methods available for enriching genomic libraries (Cronn et al., 2012), though most are optimized for short rather than long inserts. Possibilities include enriching for low-copy number genes based on melting kinetics (Yuan et al., 2003) or methylation (Palmer et al., 2003) or using hybridization against expressed genes (Lovett et al., 1991; Fu et al., 2010). While further method development would be needed, there is abundant scope for generating transgenomic libraries where a far higher proportion of clones contain causally important genes, thereby making screens dramatically more efficient.

## Prospects For Transgenomics

Transgenomics has great potential for contributing to the study of gene function in genetic model species and for identifying the molecular changes that underlie phenotypic evolution. While transgenomic screens will require a significant investment of effort and money, evolutionary theory and data from candidate gene transformation experiments show that, at least in Brassicaceae, the costs may well be outweighed by the fundamental data that will be obtained.

The initial ventures in transgenomics should aim to answer some important outstanding questions. In particular it is critical that an effort be made to quantify the proportion of a genome that causes different kinds of dominant phenotypes (morphological changes, sterility, lethality, etc.) as a function of phylogenetic distance between donor and recipient species. Further, there would be great value in studying two closely related species and conducting a complete, reciprocal transgenomics screen in order to determine the total number of major genes responsible for their different phenotypes. Lastly, we believe it would be beneficial for at least the *Arabidopsis* community to invest in an ordered transgenomics resource deposited in stock centers. If sets of sequenced clones from different donor species were each associated with transgenic seed, a researcher interested in a particular gene family could order up seed containing exogenous versions of that gene to look for phenotypes that may reveal aspects of gene function. Similarly, a scientist seeking new genes involved in the development of a phenotype could identify a donor and recipient species pair that differ in the phenotype and then could screen the corresponding transgenomic lines.

Looking to the future, it seems likely that transgenomics will emerge as an important tool for basic plant research. It is also possible that transgenomics could have applied importance. As a complement to traditional mass selection and candidate gene genetic modification, breeders may find it useful to introduce genetic variation in bulk from foreign species followed by screening and selection based on desirable traits that emerge. For all these reasons, we hope that this article will stimulate efforts to develop transgenomics as a tool for plant genetic research.

## Conflict of Interest Statement

The authors declare that the research was conducted in the absence of any commercial or financial relationships that could be construed as a potential conflict of interest.
